# Should Physicians Prescribe Alcoholic Stimulants

**Published:** 1871-08

**Authors:** A. Y. McCormick

**Affiliations:** Fowler, Ill.


### SHOULD PHYSICIANS PRESCRIBE ALCOHOLIC STIMULANTS?

Fowler, Ill., *July 12th, 1871.*

Before answering this question, it will be proper to enquire into the relation the physician sustains to his patient and to the community, as well as into the nature and effects of alcohol.

The physician's profession is devoted exclusively to the welfare of mankind, and is physico-moral in its mode of investigation and action.

The physician has to deal with man in his highest nature, as well as in his physical. The exhibition of prescriptions is not his only work; it is merely an adjunct. He is not detailed simply to superintend a course of treatment, but to stand as the custodian and conservator of the highest interests and most precious blessings conferred upon our race.

The omnipotent fiat has gone forth, "The *soul that sinneth, it shall die.*" So sin brought death, and death brings woe and anguish. The physician's mission is to lessen this woe and lighten this curse, and it at once assumes the grandest proportions. The physician is not only made the special guardian of human life, but also the guardian of true science and sound principles. A leading object with him should be the deduction and establishment of great general principles, from a comparison of the results of his own observation with the accumulated experience of all ages.

What, then, from observation and experience, do we find to be the nature and effect of alcohol?

All scientific investigation has invariably led to the logical conclusion that alcohol is essentially a poison—precisely as arsenic, opium, and nicotine are poisons; that the difference between a liquor containing five per cent and one containing fifty per cent of alcohol, is one of degree merely—not of kind—at least so far as poison is concerned.

Since alcohol is a poison, it is evident to any unprejudiced mind, that even a very limited use in health of anything containing it would be productive of evil; because, if much poison will do great harm, a little poison must do some harm; but, as physicians prescribe other poisons in disease, may they not prescribe alcohol? We think not, for two reasons.

1st. In those cases where alcoholics are used, there are other agents which will answer the purpose as well, or better, and, 2d. The prescription of alcoholics, even where they appear to be indicated, is evil in its tendency (because the desire for narcotics—as alcohol—increases with their use), and physicians, being necessarily admitted to the sanctum of domestic life, are morally prohibited from introducing within its pale any such "deceiver" and "mocker" as alcohol, without an absolute necessity, and we believe such necessity never exists.

All the medicinal properties ascribed to alcohol by the advocates of its prescription are those of antiseptic, anæsthetic, and stimulant. If we wish to prescribe an antiseptic, we have carbolic acid and others, which will answer the purpose better. If we wish to prescribe anæsthetics, we have far better and more harmless ones in ether and chloroform. If we wish to prescribe stimulants, we have more effective and more innocent ones in ginger, carbonate of ammonia, etc.

The erroneous practice of flying to the whisky bottle, by the masses, as a panacea, has grown out of the medical prescription of alcoholics, and will never be remedied until physicians cease to prescribe them.

As long as the public regard alcoholics, in themselves, good (which they are not), so long will the fruitage be found in broken hearts and desolate homes. Physicians, as members of a most liberal and philanthropic profession, owe it alike to themselves and to their fellow-men, to adopt the more scientific view—that alchoholics are unnecessary at all times and under all circumstances—which is acted upon already by many of the leading lights of the profession, and interdict the use of alcoholics under all circumstances.

A. Y. McCormick, M.D.

